# Reconstruction of a dorsal thoracic wall defect with a dorsal intercostal artery perforator flap after removal of a bulky cutaneous squamous cell carcinoma: a case report

**DOI:** 10.1186/s13256-019-2226-1

**Published:** 2019-09-17

**Authors:** E. Lupon, A. G. Lellouch, F. Deilhes, B. Chaput, C. Berthier

**Affiliations:** 1 Department of Plastic Reconstructive Surgery and Burns, Rangueil University Hospital, 1 avenue du Professeur Jean Poulhès, 31400 Toulouse, France; 2 Department of Plastic Reconstructive Surgery, European Georges Pompidou Hospital (AP-HP), Paris, France; 3 Department of Plastic Surgery, Massachusetts General Hospital/Harvard Medical School, Boston, MA USA; 4 Department of Dermatology, Larrey University Hospital, Toulouse, France

**Keywords:** Carcinoma, Squamous cell, Perforator flap, Surgical flaps

## Abstract

**Introduction:**

Surgical reconstruction of large soft tissue defects of the upper back is challenging. Although the usefulness of free perforator flaps has been demonstrated, local options remain limited. The dorsal intercostal artery perforator flap was recently described but its use is still uncommon.

**Case report:**

An 88-year-old Causasian woman presented with a large, ulcerated, left prescapular cutaneous squamous cell carcinoma (T3N0M0). Complete excision was performed, and the resulting defect was reconstructed with a dorsal intercostal artery perforator flap based on two perforators. Postoperative recovery was uncomplicated and adjuvant radiotherapy commenced 10 weeks later.

**Conclusion:**

Compared to conventional muscle flaps, the dorsal intercostal artery perforator flap offers greater protection of muscle function, is less invasive, and lowers donor site morbidity. Based on these advantages, this flap should be considered a useful local option for reconstructing large cutaneous defects of the upper back.

## Introduction

The standard of care in cutaneous squamous cell carcinoma (SCC) is surgical excision. Depending on body location, defect coverage can be challenging for the reconstructive surgeon. Perforator flaps are useful, particularly in post-oncologic resections, as they allow reduced morbidity of donor sites and tailoring of flap design according to the extent of the resultant defect [1, 2]. The dorsal intercostal artery perforator (DICAP) flap was recently described but clinical application remains limited [3]. DICAP flaps have several advantages compared to muscle and muscle–skin flaps, such as the preservation of muscle functions, less invasiveness, and lower donor site morbidity. The DICAP flap has a high capacity for mobilization. Therefore, it can be used to repair all back defects, especially median and paramedian defects. Based on these advantages, we suggest that this flap should be considered a useful option for the repair of back defects, even of large size.

Here, we describe the reconstruction of a large, upper back cutaneous defect with a local DICAP flap with low donor site morbidity, authorizing adjuvant radiotherapy in the area.

## Case presentation

**Fig. 1:**
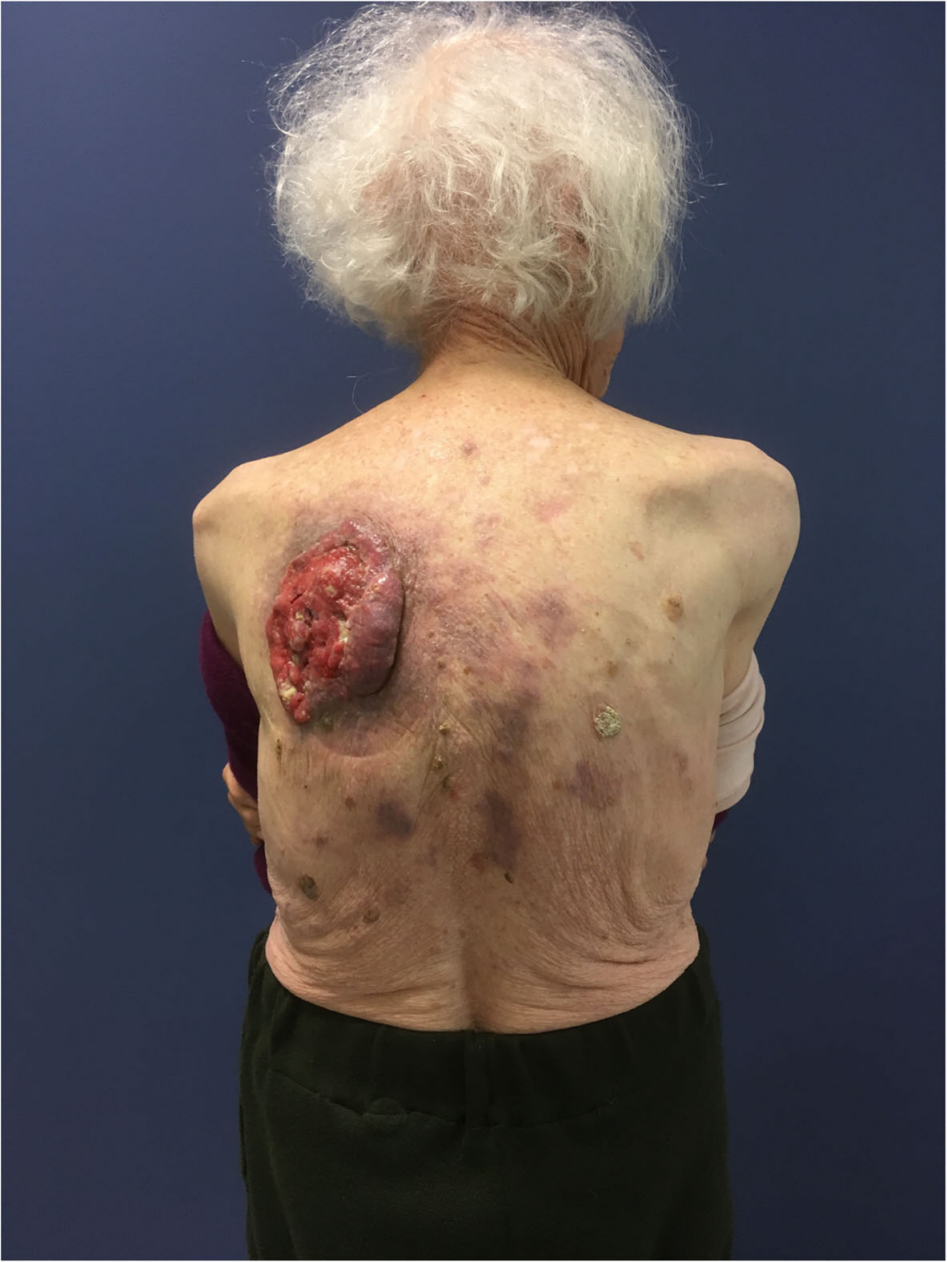
Preoperative dorsal view of the left prescapular cutaneous squamous cell carcinoma

**Fig. 2:**
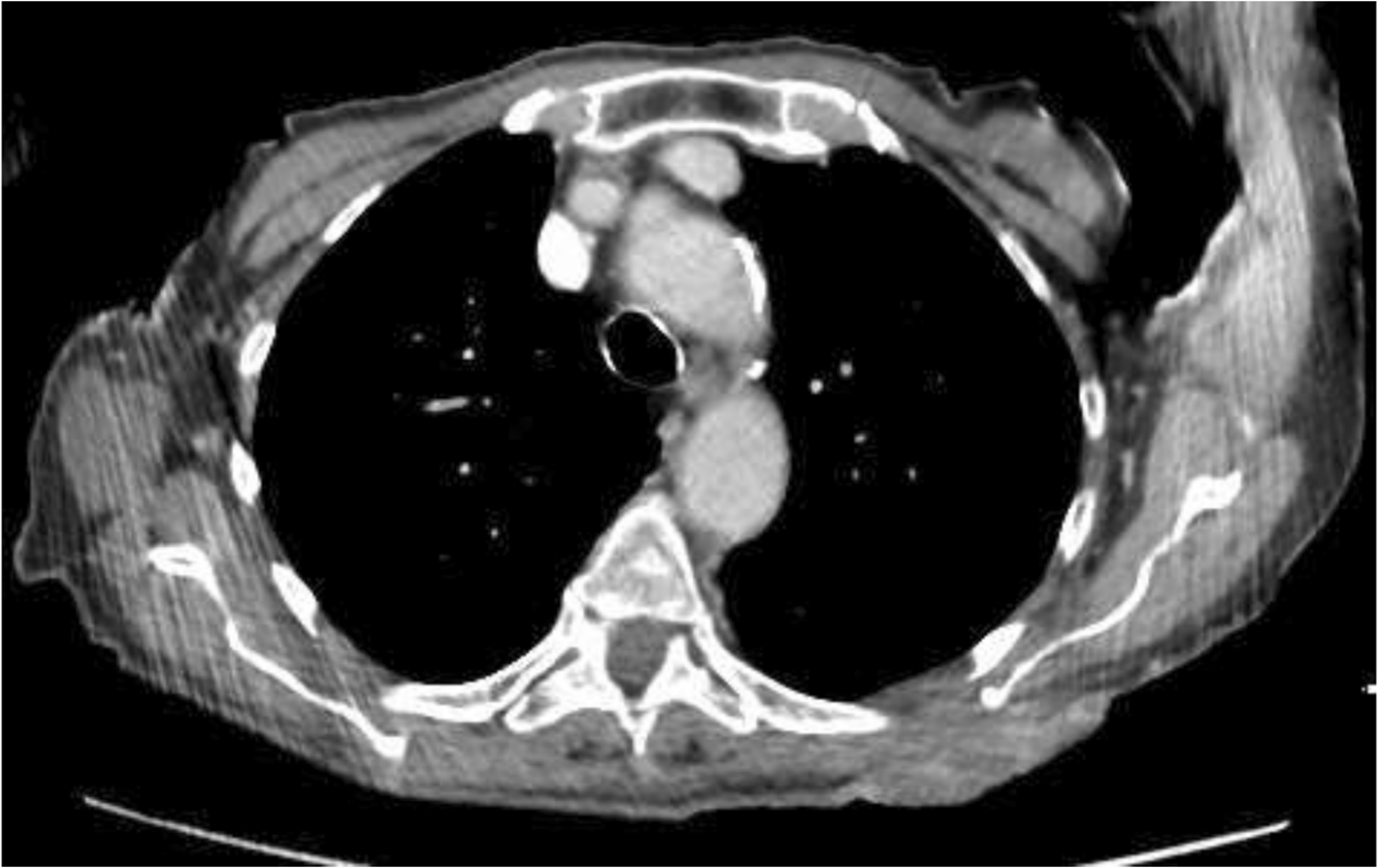
Thoracic computed tomography in axial section showing superficial dorsal lesion with no invasion of the scapula at depth

An 88-year-old Causasian woman presented with a large mass at the left prescapular region that had progressed in size over several months. Two years prior, she had undergone surgical excision of a SCC at the same site but resection margins were inadequate. She refused further treatment, was lost to follow-up and has been on homeopathy since. She is otherwise healthy with no significant comorbidities and does not smoke tobacco or drink. In fact, she had no attending physician and suffered from no pathology. She was rather resistant to medical treatment and had consulted very few doctors during her lifetime. Thus, she did not take any treatment except homeopathy to keep her in good general shape. She had no other medical history other than the surgical cure of a bladder prolapse and a right native hip luxation which was reduced in an operating room a few months before our first consultation. Socially, she is a retiree from laboratory work, who has been widowed for 5 years, and lives alone with three adult children nearby. She had a normal neurological examination at the consultation. On physical examination, she appeared cachectic and had received food supplements during hospitalization and there were numerous dermatological lesions suspected of being skin tumors. She had a heart rate of 78 beats per minute; her blood pressure was 125 mmHg (systolic) and 75 mmHg (diastolic). Her temperature was normal (37.6 °C). No medication was prescribed prior to surgery. However, on this physical examination, there was a notable fungating mass (measuring 10 cm in its major axis) involving the deeper tissues with contact bleeding (Fig. 1). SCC was confirmed on biopsy. Oncological work-up included ultrasonographic evaluation of draining lymph nodes in the axillae and groin bilaterally, and a staging thoracoabdominal computed tomography (CT) scan (Fig. 2); these were negative. Final staging of the SCC was T3N0M0 according to the 8th edition of the American Joint Committee on Cancer (AJCC) Classification [4] and was considered high risk due to size and recurrence [5]. Surgical resection with 1 cm margins and postoperative adjuvant radiotherapy was recommended following multidisciplinary discussion. Hence, safe and reliable soft tissue coverage would be required.

**Fig. 3:**
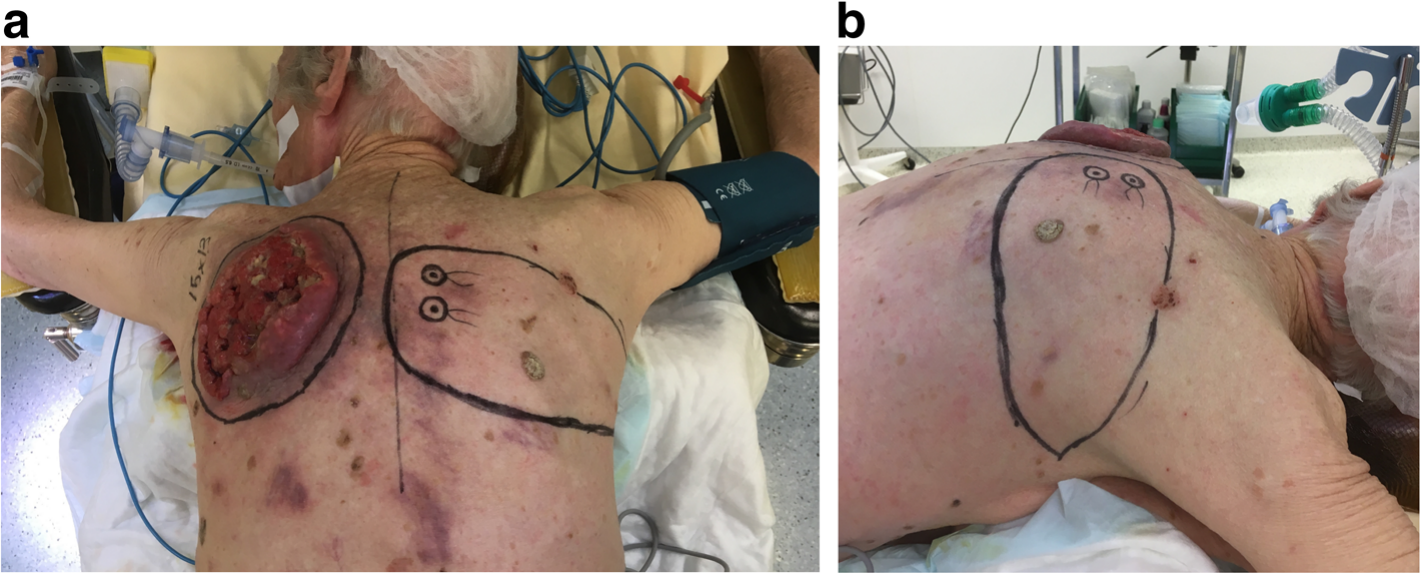
Preoperative planning of the oncological resection and design of the dorsal intercostal artery perforator flap. **a** Coronal view. **b** Lateral view

**Fig. 4:**
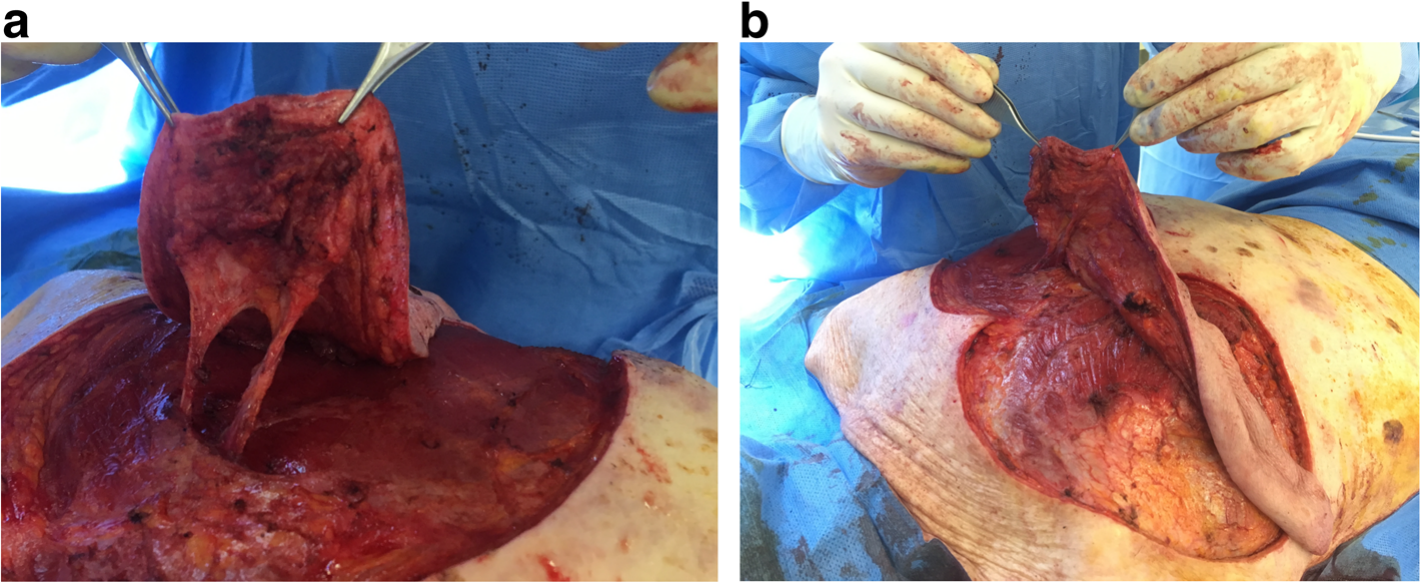
Intraoperative view of the two perforators of the dorsal intercostal artery perforator flap. **a** After skeletonization of the perforators. **b** After flap rotation prior to inset

**Fig. 5:**
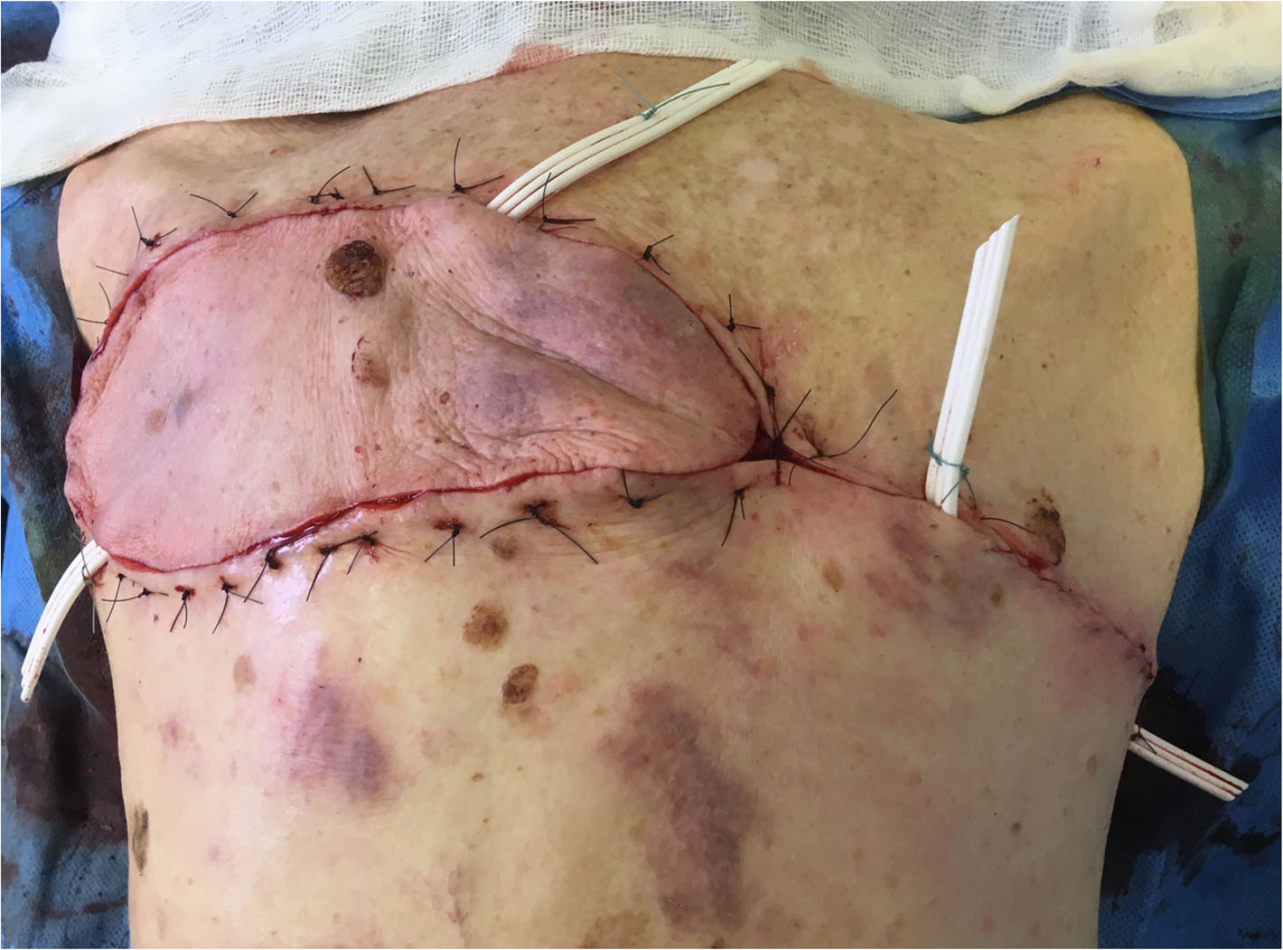
View of the reconstruction at the end of the surgery

**Fig. 6:**
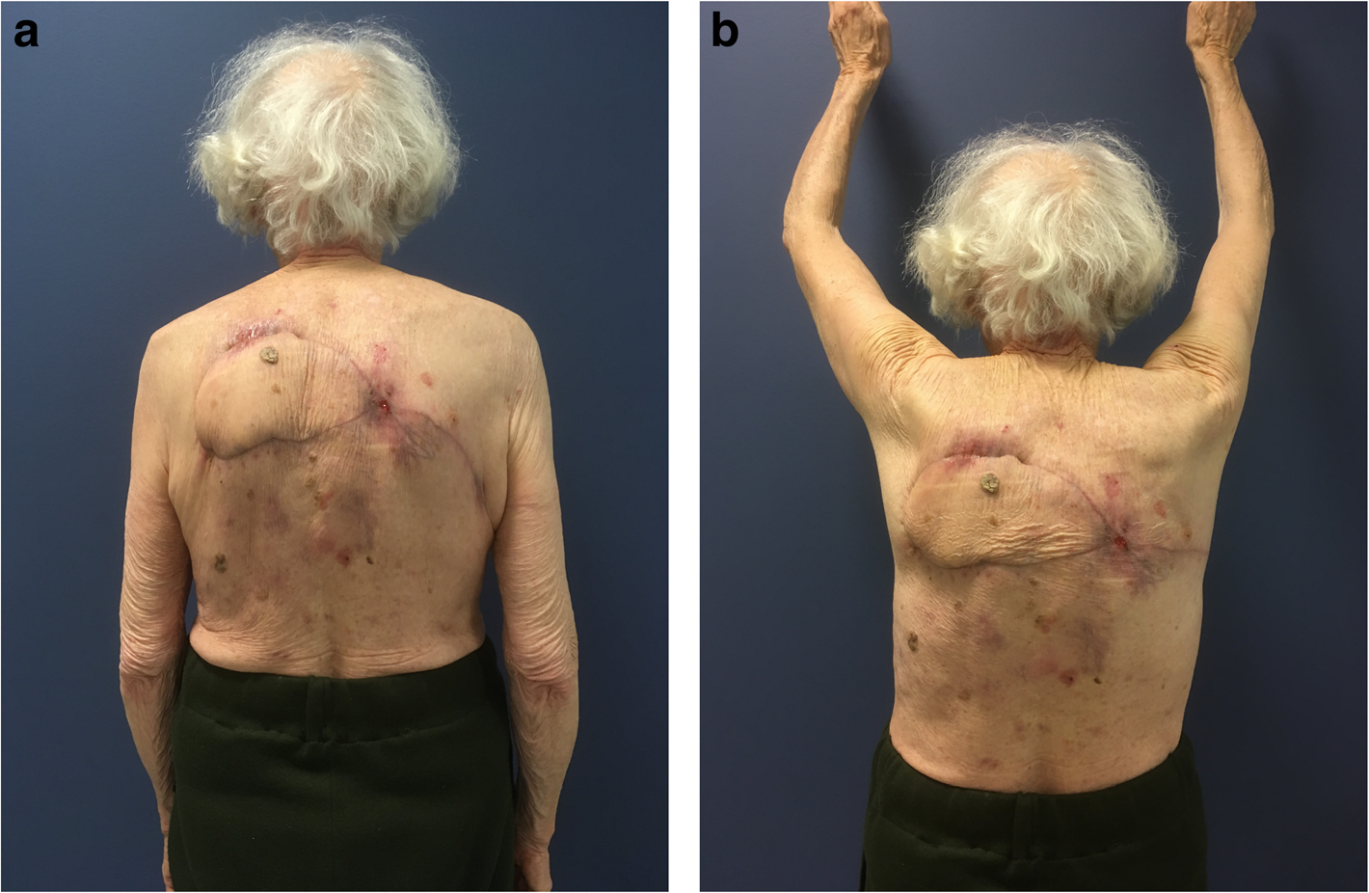
Postoperative dorsal view of the reconstruction by the dorsal intercostal artery perforator flap. **a** At rest. **b** With full abduction of arms

Oncologic resection was performed with clear separation obtained intraoperatively between the lesion and underlying deep structures. A DICAP flap was then harvested from the contralateral upper back (Fig. 3): two perforators were identified using a handheld Doppler (Fig. 3a) and the flap was designed to extend laterally to obtain as much skin as possible, with a final skin paddle of 17 × 9 cm (Fig. 3b). Flap dissection then proceeded from distal to proximal in a subfascial plane. The two perforators were skeletonized (Fig. 4) to minimize pedicle kink and enable 180º rotation into the post-resection defect. The flap and donor site were then closed over suction drains (Fig. 5). Venous congestion was observed immediately after positioning the flap over the post-excision defect site, but this resolved spontaneously in 15 minutes. Postoperatively, our patient was instructed against supine positioning to protect the flap from overlying pressure for 5 days. Surgical drains were removed on postoperative day (POD) 5. During hospitalization, she benefited in the immediate postoperative period from a volemic expansion by 500 mL of polyionic 5% polyionic as well as anti-emetics (ondansetron 4 mg/2 ml) and painkillers of grade 1 (paracetamol 1 gram) and grade 2 (ketoprofen 50 mg), initially by intravenous and then by oral route. Supplementation with food supplements was introduced (twice a day). In order to prevent thromboembolic complications, preventive anticoagulation was implemented with a low molecular weight heparin called Lovenox (enoxaparin) 0.2 IU. Gastric protection with pantoprazole 20 mg was introduced for a few days. She had a small postoperative anemia that was treated with orally administered iron called Tardyferon (ferrous sulfate) 80 mg. Her cell blood count was normal except for mild regenerative anemia. Her renal and hepatic function was also without particularity. No urinary or microbiological analysis was carried out during hospitalization. Wound healing was complete at POD 21 except for two small areas of minor wound dehiscence < 2 cm and 3 cm at the lateral and superior edges of the flap, respectively, due to slight tension on closure. The former healed by secondary intention but the latter required debridement and closure under local anesthesia. The final postoperative appearance was acceptable to our patient (Fig. 6a) and she did not have functional limitation of her arms (Fig. 6b).

A histopathological examination revealed a well-differentiated, invasive cutaneous SCC, of the common type, measuring 100 × 20 mm with Clark V level of invasion without neurovascular involvement. Final excision margins were uninvolved at > 9 mm laterally and > 1 mm posteriorly. Due to the large initial tumor size, local recurrence, and close posterior margin of 1 mm, adjuvant radiotherapy was initiated at 10 weeks postoperatively. Surveillance with biannual dermatological and ultrasonographic assessments was arranged. At 6-month follow-up, she was clinically well with no evidence of locoregional or distant SCC recurrence and the flap had completely healed with no bother to our patient.

## Discussion

Building on anatomical studies of the DICAP flap [3], our case report has demonstrated its clinical application in large upper back soft tissue coverage and its ability to tolerate further radiotherapy without sequelae. In our case, an individualized, one-stage treatment plan was devised because of our patient’s age (leading to anesthetic and surgical limitations in terms of postoperative rehabilitation) and her preference for alternative treatments which would have compromised staged surgeries requiring daily dressing care [6].

Various reconstructive options exist for coverage of upper back cutaneous defects. While amenable to skin grafting, the need for postoperative adjuvant radiotherapy in our case precluded its use and a more reliable and robust coverage would be necessary, such as a musculocutaneous flap. Such local options include the parascapular, trapezius, and latissimus dorsi flaps but the proximity of their edges to the resection margins would, in turn, limit the size of the skin paddle that could be harvested. Free flap transfers with anastomoses to the axillary vessels were also considered but require longer operative times and a prolonged hospital stay that do not suit elderly patients such as ours. Therefore, it became readily apparent that a local, fasciocutaneous perforator flap would be the best option given the shorter operative times (compared to free tissue transfers) and decreased operative morbidity (compared to muscle flaps).

With improved knowledge of the cutaneous vasculature, perforator flaps are increasingly used in clinical practice to minimize the morbidity of donor sites. They also afford much flexibility in design, facilitated by the use of a handheld Doppler ultrasound to identify specific cutaneous perforators, and can be adapted to the dimensions required at the intended recipient site. These qualities render perforator flaps as an ideal reconstructive tool and are now often used to manage and cover various cutaneous defects [1, 2]. Building on these, the propeller flap represents a type of local perforator flap that, according to the “Tokyo Consensus,” is defined as “an island flap that reaches the recipient sites through an axial rotation” [7]. Hyakusoku *et al.* first used the term “propeller flap” in 1991, describing two subcutaneous pedicled island flaps, vascularized by a perforator artery in the center and rotated 90°, for the reconstruction of skin scar contractures in burn patients [8]. In this case, we were able to utilize a local DICAP flap based on two perforators as a propeller flap and achieved soft tissue cover in 2.5 hours for our 88-year-old patient.

The DICAP flap is vascularized by the intercostal perforator arteries [1, 3, 9, 10]. In 1978, Daniel *et al*. described the vascular anatomy of the posterior intercostal arteries [11]. The posterior intercostal artery (PICA) is divided into four segments: vertebral, costal, intermuscular, and rectus, based on the neurovascular branching pattern. The flaps based on the perforators of dorsal, dorsolateral, and lateral branches of the PICA and anterior intercostal branches of the internal mammary artery are termed DICAP, dorsolateral intercostal artery perforator (DLICAP), lateral intercostal artery perforator (LICAP), and anterior intercostal artery perforator (AICAP) flaps, respectively. DICAP are constant vessels located laterally at less than 5 cm from the front of vertebral spines, giving a wide perforasome and allowing flaps to be harvested with a maximum skin paddle of 40 cm × 15 cm [12]. Choke anastomoses are present between the dorsal rami of PICA, the musculocutaneous perforators of latissimus dorsi, lateral rami of PICA of the adjacent intercostal spaces, and with the circumflex scapular and thoracodorsal arteries. Many anatomical variations can exist in this region and a preoperative Doppler is highly recommended for localization of the perforator. Its reported clinical uses include post-tumor excision coverage (neurofibroma, sarcoma, melanoma, and cutaneous carcinoma), closure of myelomeningocele, coverage of exposed spinal hardware, and pressure sore coverage [12–16].

## Conclusion

The reliable perforator anatomy of the DICAP flap allows shortened operative times for soft tissue coverage of the upper back. We have shown in this case report that the DICAP flap allowed safe and durable reconstruction after oncologic excision of a large, recurrent left prescapular cutaneous SCC, to enable adjuvant radiotherapy without wound complications.

## Data Availability

The dataset used and analyzed during the current study is available from the corresponding author on reasonable request.
